# Cellular Pathology of Pelizaeus-Merzbacher Disease Involving Chaperones Associated with Endoplasmic Reticulum Stress

**DOI:** 10.3389/fmolb.2017.00007

**Published:** 2017-02-24

**Authors:** Ken Inoue

**Affiliations:** Department of Mental Retardation and Birth Defect Research, National Institute of Neuroscience, National Center of Neurology and PsychiatryKodaira, Japan

**Keywords:** unfolded protein response, hypomyelinating leukodystrophy, ER chaperone, point mutations, PLP1

## Abstract

Disease-causing mutations in genes encoding membrane proteins may lead to the production of aberrant polypeptides that accumulate in the endoplasmic reticulum (ER). These mutant proteins have detrimental conformational changes or misfolding events, which result in the triggering of the unfolded protein response (UPR). UPR is a cellular pathway that reduces ER stress by generally inhibiting translation, increasing ER chaperones levels, or inducing cell apoptosis in severe ER stress. This process has been implicated in the cellular pathology of many neurological disorders, including Pelizaeus-Merzbacher disease (PMD). PMD is a rare pediatric disorder characterized by the failure in the myelination process of the central nervous system (CNS). PMD is caused by mutations in the *PLP1* gene, which encodes a major myelin membrane protein. Severe clinical PMD phenotypes appear to be the result of cell toxicity, due to the accumulation of PLP1 mutant proteins and not due to the lack of functional PLP1. Therefore, it is important to clarify the pathological mechanisms by which the PLP1 mutants negatively impact the myelin-generating cells, called oligodendrocytes, to overcome this devastating disease. This review discusses how PLP1 mutant proteins change protein homeostasis in the ER of oligodendrocytes, especially focusing on the reaction of ER chaperones against the accumulation of PLP1 mutant proteins that cause PMD.

## Clinical and genetic basis of Pelizaeus-Merzbacher disease (PMD)

**Figure 1:**
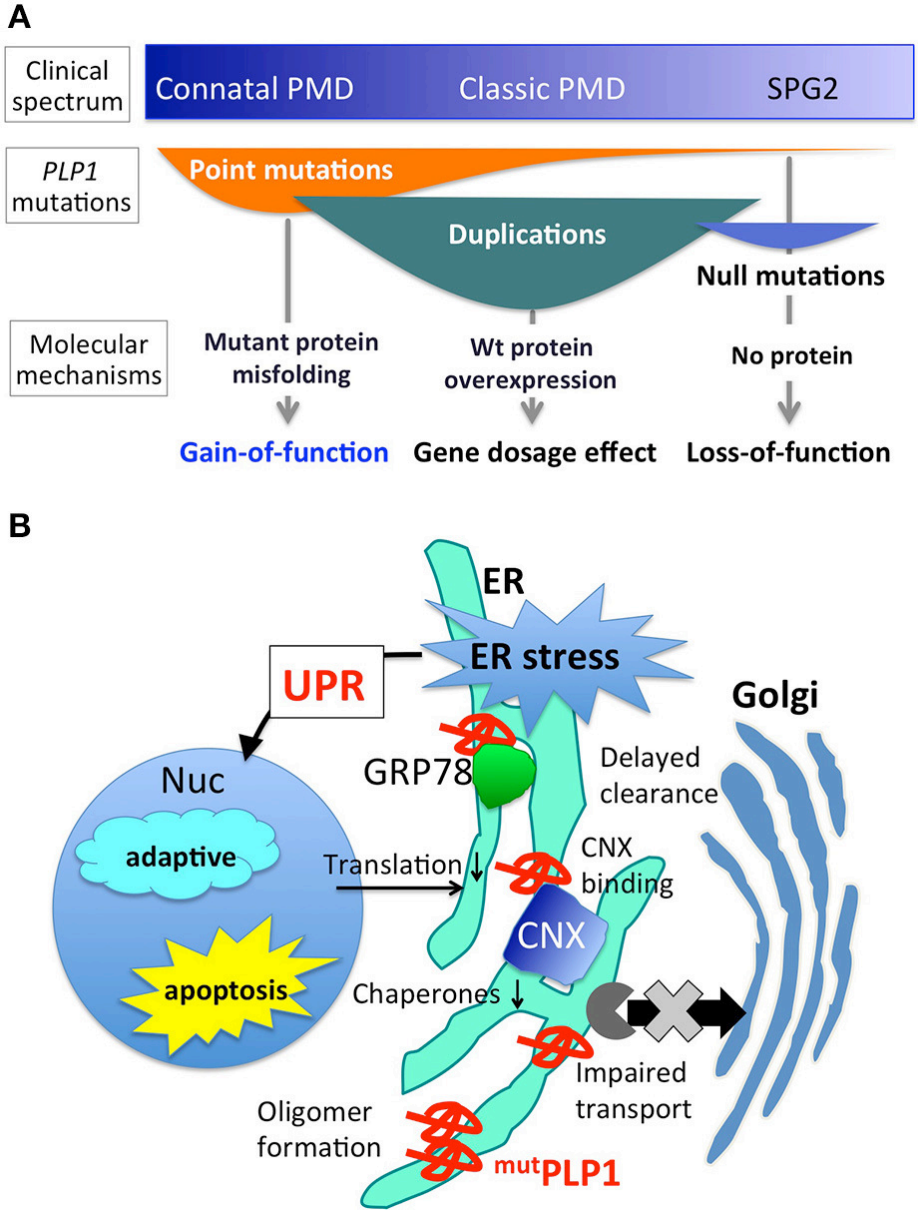
*****PLP1*** mutations, associated phenotypes, and molecular mechanisms. (A)** Different PLP1 mutations result in distinct molecular mechanisms underlying a wide variety of clinical phenotypes (Inoue, 2005). **(B)** Mutant PLP1 and associated cellular pathology. Misfolded mutant PLP1 (^mut^PLP1) accumulates in the ER and evokes ER stress, which triggers unfolded protein response to rescue the cells by reducing translation and increasing ER chaperones, or to turn on the proapoptotic pathway to induce cell death (Southwood et al., 2002; D'Antonio et al., 2009; Clayton and Popko, 2016). Disease-causing mutations in PLP1 is known to cause oligomer formation in the ER (Swanton et al., 2005), binding to CNX and delayed clearance from the ER (Swanton et al., 2003), and impaired ER-Golgi trafficking (Numata et al., 2013).

Pelizaeus-Merzbacher disease (PMD) is a pediatric inherited disorder of the central nervous system (CNS), mainly affecting oligodendrocytes, cells specialized in generating and maintaining myelin sheaths. Myelin is a membranous structure that wraps around the neuronal axons, enhancing the electronic conduction of neuronal circuits in the brain. Myelin also enables rapid and efficient movement of the body and cognitive function of the brain. Children with PMD thus show deficiencies in motor and intellectual development at an early stage of life, which usually continue thereafter. In addition, they present other neurological symptoms including nystagmus (involuntary rapid eye movement), spastic paraplegia (increased muscle tone and stiffness), ataxia (abnormal voluntary coordination of muscle movements), and dystonia (involuntary muscle contractions). PMD shows a wide range of clinical severity (Figure 1A). The most severe cases (the connatal form) show the arrest of developmental milestones such as head control, and are often bedridden for their lifetime. Milder cases (the classic form) reveal delayed motor and cognitive development with different degrees; for example, some patients obtain the ability to walk independently or achieve head control, but are wheelchair-bound. In general, motor disability is more severe than cognitive dysfunction. The mildest cases display spastic paraplegia with mild cognitive impairment, often diagnosed as spastic paraplegia type 2 (SPG2). The incidence of PMD with *PLP1* mutations was estimated to be 1.45 and 1.9 per 100,000 male live births in Japan and USA, respectively (Bonkowsky et al., 2010; Numata et al., 2014).

The underlying cause of PMD is either an abnormal quality or quantity of the proteolipid protein 1 (PLP1), which is the most abundant myelin membrane lipid protein in the CNS (Inoue, 2005). *PLP1* is located at Xq22.1 on the long arm of the X chromosome and encodes tetra-span myelin membrane lipoprotein; hence PMD shows X-linked recessive pattern of inheritance. Two alternative splicing variants differ in the inclusion or exclusion of the latter half of exon 3, to produce either PLP1 or DM20 protein; the former composes the major portion in the mature myelin. (Griffiths et al., 1998; Yool et al., 2000).

Different *PLP1* mutations cause PMD through distinct molecular mechanisms (Figure 1A). Point mutations in the coding exons often lead to amino acid substitutions that alter protein conformation, resulting in a misfolded protein (Jung et al., 1996; Dhaunchak et al., 2011). Approximately 30–40% of PMD patients worldwide have point mutations in their *PLP1* gene (Numata et al., 2014). This review focuses on the molecular mechanisms underlying this class of mutations. Genomic duplication events of *PLP1* also cause the PMD phenotype (Inoue et al., 1996, 1999), due to the overexpression of the *PLP1* transcript. However, the exact cellular mechanism as to how an extra copy of the wild-type *PLP1* gene leads to a severe hypomyelinating phenotype, remains unknown. Duplication of the *PLP1* gene is the most common mutation that causes the PMD phenotype, since 60–70% of PMD patients have it and this proportion appears to be quite similar worldwide (Inoue, 2005; Numata et al., 2014). Rare *null* mutations, such as gene deletions or nonsense/frame shift mutations that result in premature terminations (presumably degraded by nonsense mediated mRNA decay) leading to no PLP1 production can cause a mild but slowly progressive PMD phenotype (Inoue et al., 2002; Garbern, 2007). Intronic and splicing mutations have been found in a considerable amount of patients, who also show variable PMD phenotype severity (Hobson et al., 2000, 2002; Laššuthova et al., 2013; Kevelam et al., 2015). Each of these mutations is associated with a specific clinical phenotype of PMD, as detailed in a previous review (Inoue, 2005).

Many *PLP1* point mutations cause amino acid substitutions, leading to the production of misfolded PLP1 that accumulates in the endoplasmic reticulum (ER) (Gow and Lazzarini, 1996; Swanton et al., 2005). In humans, production of wild-type PLP1 rapidly increases upon the maturation of oligodendrocytes in the process of myelination to produce massive amounts of myelin after birth. The secretory system runs at full capacity in the maturating oligodendrocytes to produce both myelin proteins and lipids. Therefore, in PMD patients, a large amount of PLP1 mutant proteins accumulates in the ER of oligodendrocytes, eventually leading to apoptotic cell death and myelination failure; however, the exact pathological mechanism is currently unknown. Mutant PLP1 proteins do not form aggregates or insoluble amyloid-like structures, but they form SDS-resistant homo oligomers, which is more prominent in mutations associated with severe clinical phenotype (Swanton et al., 2005).

## ER stress and unfolded protein response (UPR)

**Figure 2:**
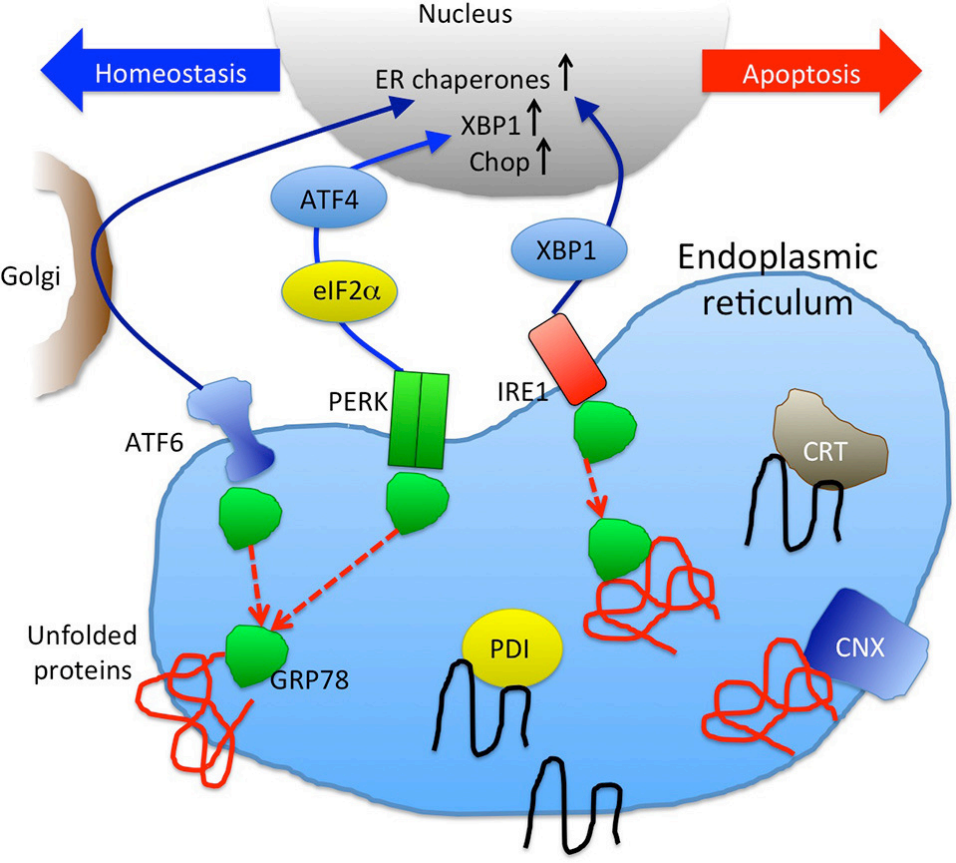
**A schematic picture of unfolded protein response and ER chaperones**. GRP78 binding to unfolded proteins accumulated in ER results in dissociation from and activation of ER stress sensors, ATF6, PERK, and IRE1 (Szegezdi et al., 2006). Downstream signaling molecules function either to maintain cellular homeostasis or promote apoptosis. UPR result in increase of ER chaperones (CRT, CNX, GRP78, and PDI are depicted here) to enhance the protein folding capacity.

Recent studies have revealed that mutant PLP1 may cause PMD, not by lack of functional protein, but by eliciting a cytotoxic effect (Schneider et al., 1995; Swanton et al., 2005; Numata et al., 2013). Especially, unfolded protein response (UPR) has been suggested to play a central role in the molecular pathology of PMD (Figure 2; Southwood et al., 2002; D'Antonio et al., 2009; Clayton and Popko, 2016). Regardless of the cell's pathological or physiological state, unfolded/misfolded proteins accumulate in the ER, causing ER stress; hence, these aberrant proteins need to be removed from the ER to maintain cellular homeostasis. The cellular signaling cascade that plays this role is the UPR, a stress-induced eukaryotic signaling cascade that serves as a cellular quality control (Walter and Ron, 2011). UPR protects cells from the toxicity of accumulated proteins in the ER by reducing translation, increasing retrotranslocation and degradation of ER-localized proteins, and bolstering the protein folding capacity of the ER to maintain cellular homeositasis. However, when the ER stress exceeds the capacity of this intrinsic quality control, apoptosis is induced through the up-regulation of the proapoptotic branch of the UPR pathway (Lin et al., 2007).

UPR activation is triggered by three distinct pathways: ATF6, IRE1, and PERK (Szegezdi et al., 2006) (Figure 2). These pathways are negatively regulated by GRP78 (also known as BiP), an ER chaperone that plays a critical role in the initiation of UPR (Schröder and Kaufman, 2005). When unfolded/misfolded proteins accumulate in the ER, GRP78 binds to these unfolded/misfolded proteins leading to the dissociation of the ER stress sensors (i.e., ATF6, IRE1, and PERK), thereby triggering UPR. ATF6 induces the transcription of major ER chaperones and XBP1 (Yamamoto et al., 2007). The endonuclease activity of IRE1 promotes the splicing of *XBP1* mRNA, producing its active form that encodes a transcription factor that regulates the expression of UPR-related genes (Calfon et al., 2002). ATF6 and IRE1-XBP1 axes promote the expression of ER chaperones, facilitating the correct folding and/or assembly of proteins in the ER, preventing ER protein aggregation, thereby improving cell survival (Yoshida et al., 2001; Szegezdi et al., 2006; Yamamoto et al., 2007). However, when this intrinsic quality control system fails to eliminate unfolded/misfolded proteins, UPR activates a proapoptotic signaling cascade, which is initiated by the dissociation of GRP78 from PERK. Phosphorylated PERK decreases global protein translation by phosphorylating the eukaryotic initiation factor 2α (eIF2α), reducing the ER protein load, while PERK also increases the translation of some UPR-related genes including ATF4, which leads to the transcriptional activation of CHOP. CHOP is a transcription factor that induces apoptosis by directly repressing the expression of anti-apoptotic factor Bcl-2 (Hetz, 2012).

The activation of the UPR-induced apoptotic pathway, caused by the accumulation of mutant PLP1, may cause massive cell death of oligodendrocytes, which is suspected to occur in the brains of patients with PMD.

## ER stress and UPR in PMD

Clinical and genetic observations of patients with PMD have raised two questions in terms of the molecular pathology of *PLP1* point mutations. First, PMD patients with point mutations show a wide range of clinical severity; the mildest end of this clinical spectrum shows as spastic paraplegia type 2 (SPG2), which is considered to be milder disease than PMD, while the most severe end is the connatal form PMD, which can cause premature mortality (Inoue, 2005). The exact reason as to why different point mutations located in various positions of *PLP1* result in different clinical severity remains unknown. Second, it has been speculated that mutant PLP1 may act as gain-of-function proteins, because many patients with point mutations in *PLP1* show much severer phenotypes than those lacking a functional PLP1 protein, due to deletion or truncating mutations. However, the molecular basis by which mutant PLP1 elicits toxicity to oligodendrocytes remains to be determined.

The initial evidence addressing the question of cell toxicity due to the production of the mutant PLP1 was given by a cell biological study that demonstrated that the *PLP1* mutations associated with severe phenotypes led to the accumulation of both PLP1 and DM20 isoforms in the ER, while those associated with milder phenotypes resulted in the traverse of DM20 isoform through the secretory pathway to the cell surface (Gow and Lazzarini, 1996). Furthermore, the accumulation of mutant PLP1 was found to trigger ER stress and activate UPR (Figure 1B) (Southwood et al., 2002). During the maturation of oligodendrocytes, these cells produce a large amount of myelin sheath, and PLP1 abundance increases drastically. Nearly half of the entire protein content of mature oligodendrocytes is made up of PLP1. Therefore, it is not difficult to speculate that a significant amount of mutant PLP1 accumulates in the ER, causing an increased ER stress, resulting in the acute activation of the UPR apoptotic signaling cascade. In fact, CHOP, a proapoptotic transcription factor, is upregulated in the brains of patients with PMD and mouse models (Southwood et al., 2002). Upregulation of UPR was also recapitulated in the PMD patient-derived iPS cells after differentiation into oligodendrocytes *in vitro* (Numasawa-Kuroiwa et al., 2014).

The genotype-phenotype correlation in *PLP1* mutations-PMD severity has been partly explained by studies showing how different mutations trigger UPR differently. In these studies, mouse models that recapitulate the different levels of PMD severity in humans have been utilized (Yool et al., 2000). The *myelin synthesis deficient* (*msd*) mice carry an A243V mutation, which results in a very severe phenotype in both humans and mice. The *rumpshaker* (*rsh*) mice with an I187T mutation serve as a model for the mildest end of the PLP1-associated severity spectrum, SPG2. These mutants, along with the classic PMD mouse model *jimpy (jp)*, have largely contributed to the understanding of the molecular pathogenesis of PMD. The mutant PLP1 protein associated with mild PMD phenotypes appear to be cleared quickly from the ER, via the proteasomal degradation pathway and/or ER exit, while the mutant PLP1 protein associated with severe PMD phenotypes is resistant to protein degradation and/or exclusion from the ER, triggering UPR activation (Roboti et al., 2009). The position of mutations in *PLP1* also contributes to the cellular pathology that triggers UPR. For example, the retention of the mutant PLP1 in the ER that carry mutations in the extracellular domain, where two disulfide bridges are located, depends on the cysteine residues that play a critical role in protein cross-linking (Dhaunchak and Nave, 2007).

It has been suggested that mutations leading to severe PMD phenotypes may enhance the CHOP proapoptotic pathway more markedly than mild PMD phenotype-associated mutations; hence, a more severe dysmyelinating phenotype may occur (McLaughlin et al., 2007; Roboti et al., 2009). If this is the case, the genetic removal of CHOP may rescue the PMD phenotype in the mouse model. However, this scenario was not that simple. Rather than rescuing the phenotype, *rsh* mice crossed with a *CHOP* null line revealed a higher premature mortality rate and an increased number of oligodendrocyte cell death (Southwood et al., 2002). The prominent worsening of PMD phenotype in mice lacking *CHOP* shows a sharp contrast in another disease model of peripheral demyelinating neuropathy, Charcot-Marie-Tooth disease (CMT), which is caused by mutations in the *MPZ* gene. The genetic removal of *CHOP* from the CMT model mice carrying S63del *MPZ* mutation ameliorated the demyelination and apoptotic cell death of Schwann cells (Pennuto et al., 2008). The exact reasons for these opposite outcomes resulting from *CHOP* ablation in central and peripheral myelin gene mutants remain unknown. One hypothesis is that the *CHOP* downstream genes in Schwann cells (and probably most other cell types) are maladaptive and CHOP induction leads to cell death and demyelination; however, CHOP may regulate a different set of downstream genes in oligodendrocytes, which presumably function as adaptive, or protective against cell death (Southwood et al., 2002; D'Antonio et al., 2009).

Interestingly, genetic ablation of ATF3 or caspase-12, both known to function in the proapoptotic pathway downstream of *CHOP*, showed no effect on disease severity in *msd* and *rsh* mice (Sharma and Gow, 2007; Sharma et al., 2007). The findings suggest that these genes are not related to oligodendrocyte survival in *PLP1* mutant mice.

## Cellular pathology beyond the UPR

Although, the central role of UPR in the cellular pathology of PMD caused by point mutations in the *PLP1* gene is indisputable, the relationship between *CHOP* upregulation and the apoptotic cell death of oligodendrocytes in PMD should be reconsidered. In order to understand the effect of mutant PLP1 accumulating in the ER, studies beyond the UPR are required to elucidate specific characteristics of the mutant PLP1 molecule, especially its interaction with other ER proteins, such as ER chaperones.

If the activation of PERK-CHOP branch of UPR is not responsible for the oligodendrocyte cell death and dysmyelination, which is the major pathology in PMD, then, who is playing the major role? The answer to this question is still unknown, but some interesting findings suggest critical roles of ER chaperones in myelinating oligodendrocytes. Mice lacking *GRP78*, in either developing or mature oligodendrocytes, showed neurological phenotypes and dysmyelination accompanied by oligodendrocyte cell death, all of which are surprisingly similar to those observed in PMD mouse models (Hussien et al., 2015). GRP78 facilitates the proper protein folding and regulates UPR by keeping the three UPR sensors (PERK, IRE1, and ATF6) inactive. In fact, these mutant mice showed activation of PERK and ATF6, and induction of CHOP expression, suggesting that the genetic ablation of GRP78 leads to persistent activation of UPR (Hussien et al., 2015). It has not been elucidated if these drastic phenotypes are associated with a perturbed physiological function of GRP78, including protein folding or induction of CHOP-mediated apoptosis.

Another example showing the critical role of ER chaperones in myelination was demonstrated by deleting calnexin (CNX) in mice (Kraus et al., 2010). CNX is a lectin-like chaperone, and together with calreticulin (CRT), promotes the folding of glycosylated proteins (Caramelo and Parodi, 2015). Despite its critical role in quality control of the secretory pathway, the genetic ablation of CNX resulted in surprisingly limited peripheral and central myelin phenotypes. CNX-deficient mice are viable, with no discernible effects on other systems, presumably because of the functional redundancy of CRT. However, in myelin systems, the mice showed apparent dysmyelination in both CNS and PNS, indicating that CNX is essential for proper myelin formation (Kraus et al., 2010). At this point, it is not clear if CNX has unique functions in myelinating oligodendrocytes and Schwann cells, or increasing the protein load in the ER during the myelination process causing insufficient folding capacity by only CRT.

Mutant PLP1 not only triggers UPR but also changes the dynamics of ER chaperones. Although, PLP1 is not a glycosylated protein and normally is not a substrate for CNX/CRT, CNX, but not CRT, stably binds to the misfolded PLP1 mutant protein (Swanton et al., 2003). Surprisingly, this CNX-mutant PLP1 binding delays the elimination of mutant protein from the cells through the ER-associated degradation (ERAD), suggesting that this glycan-independent binding of ER chaperones possibly contributes to the pathology of PMD.

CNX may not be the only chaperone involved in the pathology of PMD. Our *in vitro* study showed that mutant PLP1 reduces the ER localization of GRP78, CRT, and PDI, but not CNX (Numata et al., 2013). The exact mechanism for this apparent reduction of ER chaperones from the ER, while the ER is under stress induced by the massive production of misfolded mutant PLP1, is unknown. It has become apparent that the mutant PLP1 not only accumulates in the ER but also inhibits the transport of other proteins in the secretory pathway from ER to the Golgi apparatus. This includes the KDEL receptor, which plays a critical role in carrying ER chaperones that contains the KDEL sequence (i.e., GRP78, CRT, and protein-disulfide isomerase (PDI), but not CNX) from the Golgi apparatus back to the ER after post-translational modifications. These findings highlight the effect of mutant PLP1 on global transport of secretory proteins.

## Implication for potential PMD treatment

Considering the evidence in terms of the involvement of UPR and ER chaperones in the molecular pathology of PMD caused by mutant PLP1, some therapeutic interventions targeting the UPR and ER chaperones have been evaluated using cellular and animal models.

Curcumin, a polyphenol dietary compound derived from curry spice turmeric, has been evaluated for its therapeutic potential in some diseases in which ER stress and UPR have been implicated in the pathogenesis, such as cystic fibrosis (targeting *CFTR*), Charcot-Marie-Tooth disease (targeting *PMP22* and *MPZ*), retinitis pigmentosa (targeting *RHO*), and PMD (Egan et al., 2004; Khajavi et al., 2005, 2007; Vasireddy et al., 2011; Yu et al., 2012). *Msd* mice treated with oral curcumin showed an extended life span and a reduced number of apoptotic oligodendrocytes, showing an apparent but modest therapeutic effect. However, the expression of ER stress markers including CHOP, GRP78, myelin protein MBP, and motor function showed no change or improvement (Yu et al., 2012). Interestingly, curcumin also improved the PMD phenotype in PLP1 transgenic mouse, a model for *PLP1* duplication in patients with PMD (Epplen et al., 2015). Since the PLP1 overexpression does not involve ER stress or UPR, curcumin may also have different therapeutic targets other than UPR.

Chloroquine, an anti-malarial drug, was found to reduce the accumulation of the mutant PLP1 and attenuate ER stress by enhancing the phosphorylation of eIF2α (Morimura et al., 2014). Despite its drastic effects as an ER stress attenuator, the concentration to obtain this effect *in vitro* was relatively high (100 μM) for clinical application, considering its side effects.

Although, there is no clinically validated therapy for PMD to date, clarification of the molecular mechanism underlying the accumulation of the mutant PLP1 in the ER and its effect on cellular function and survival of oligodendrocytes, will help to identify the molecules that could rescue the cellular PMD phenotype. This could eventually lead to future treatment of patients with PMD. Enhancement of ER chaperones may serve as one possibility for the discovery of an effective treatment strategy for PMD.

## Author contributions

The author confirms being the sole contributor of this work and approved it for publication.

## Funding

This work was supported in part by research grants from Japan Agency of Medical Research and Development, AMED (Practical Research Project for Rare/Intractable Diseases: 16ek0109016) and Grant-in-Aid for Scientific Research (Kiban-B: 16H05361).

### Conflict of interest statement

The author declares that the research was conducted in the absence of any commercial or financial relationships that could be construed as a potential conflict of interest.
